# Mapping, framing, shaping: a framework for empirical bioethics research projects

**DOI:** 10.1186/s12910-019-0428-0

**Published:** 2019-11-27

**Authors:** Richard Huxtable, Jonathan Ives

**Affiliations:** Centre for Ethics in Medicine, Population Health Sciences, Bristol Medical School, Canynge Hall, 39 Whatley Road, Bristol, BS8 2PS UK

**Keywords:** Empirical bioethics, Methodology, Methods, Reflective equilibrium, Applied ethics, Integrated ethics

## Abstract

**Background:**

There is growing interest in the use and incorporation of empirical data in bioethics research. Much of the recent focus has been on specific “empirical bioethics” methodologies, which attempt to integrate the empirical and the normative. Researchers in the field are, however, beginning to explore broader questions, including around acceptable standards of practice for undertaking such research.

The framework:

In this article, we further widen the focus to consider the overall shape of an empirical bioethics research project. We outline a framework that identifies three key phases of such research, which are conveyed via a landscaping metaphor of Mapping-Framing-Shaping. First, the researcher maps the field of study, typically by undertaking literature reviews. Second, the researcher frames particular areas of the field of study, exploring these in depth, usually via qualitative research. Finally, the researcher seeks to (re-)shape the terrain by issuing recommendations that draw on the findings from the preceding phases. To qualify as empirical bioethics research, the researcher will utilise a methodology that seeks to bridge these different elements in order to arrive at normative recommendations. We illustrate the framework by citing examples of diverse projects which broadly adopt the three-phase framework. Amongst the strengths of the framework are its flexibility, since (as the examples indicate) it does not prescribe any specific methods or particular bridging methodology. However, the framework might also have its limitations, not least because it appears particularly to capture projects that involve qualitative – as opposed to quantitative – research.

**Conclusions:**

Despite its possible limitations, we offer the Mapping-Framing-Shaping framework in the hope that this will prove useful to those who are seeking to plan and undertake empirical bioethics research projects.

## Background

For several decades, research in bioethics has seen an increased interest in using and incorporating empirical data [1, 2]. This interest manifests in myriad ways, from bioethics research using empirical data to support empirical premises in argument [3], through comment on poor or unreflective use of empirical data in argument [4], to attempts to fully integrate empirical analysis into ethical theorising [5]. This latter activity, dubbed the ‘empirical turn’ in bioethics by some [1], but increasingly simply referred to as ‘empirical bioethics’, is perhaps the most controversial and challenging, requiring the development of new methodologies that provide both practical and theoretical solutions to the problem of how to develop normative claims that are richly informed by the empirical world but that do not fall into the trap of ‘doing ethics by opinion poll’.

Some empirical bioethics might, however, be best described as an attempt to grapple explicitly with the interdisciplinary nature of the field. As Ives has argued elsewhere, some accounts of empirical bioethics are less about attempting to do something completely new, and more about finding ways to better articulate and justify methods already adopted in high quality, rigorous applied ethics [6].

Arguably, the focus of the empirical bioethics literature to date has been on interrogating and articulating the process of integration through which the empirical and the normative are combined, in some way, to generate ‘should’ or ‘ought’ conclusions. A 2015 systematic review, for example, identified 32 different methodologies that attempt to articulate an integration method [7]. More recently, a move away from this explicit focus has been demonstrated in a consensus statement from a group of European researchers, which, building on others’ attempts [8], proposes standards of practice for empirical bioethics research [9]. This consensus statement reported agreement on standards of practice about the conduct and reporting of the integration process, but also looked more broadly at, inter alia, aims, research questions and training. The focus, however, on integration and standards of practice has come at the expense of substantive engagement with overarching method and process in empirical bioethics research. Integration is just one – though certainly essential – part of an empirical bioethics research project, but we must not neglect the other elements. Empirical bioethics research, as a whole, entails much more than an integrative analysis.

Given this, there may be advantages to thinking about the research process as a whole, from start to finish, in addition to its discrete parts. We have in mind not only the different phases of a particular research project, but also the different elements (or projects) that make up programmes of research, which are intended to be brought together to answer a core set of research questions. For brevity, we will typically refer to research projects. Thinking about the project (or programme) as a whole entails thinking about what comes before and/or after the specific point in the research where we might be looking to combine the empirical with the normative. It is this specific point of integration that has arguably received most attention in the literature, and our aim here is to broaden our gaze to consider how the entire research project (or programme) might be constructed and in so doing position the question of integration, properly, as one small part of a larger research process – albeit, as we shall we, one that is definitive of empirical bioethics research.

In the Centre for Ethics in Medicine, at the University of Bristol, we have developed a framework in an attempt to articulate three broad stages of empirical bioethics research. The approach as presented here was developed by Huxtable and Ives, primarily to help articulate methods for a Wellcome Trust collaborative award, [10] but draws on many years of engagement with the question of how to articulate methods and methodology in bioethics research. In what follows, we outline this (Bristol) framework, provide examples, and consider its limitations.

We are not, here, attempting to outline any specific methodology for empirical bioethics. Rather, we are attempting to articulate, and provide illustrations of, a way to think about an empirical bioethics research project holistically, and delineate between discrete research phases – of which the empirical ethics integration comprises only one – that together can contribute to answering a complex research question.

The utility here is potentially two-fold. First, there can be value in simply having terminology that allows us to conceptualise and articulate, in a recognisable form, the structure of a research project. Indeed, when we have talked about this approach in research seminars, workshops and teaching, feedback has suggested that there is value in having terminology that allows clear delineation between the phases of research but also allows reflection on how the phases interact. Second, having the terminology allows us to look at the research process in terms of separate but interlinked phases, and reflect on what the role of a particular phase is or should be. This is the value of broadening our gaze to cover the research project as a whole, rather than just focussing on the point of integration between the empirical and the normative. That point of integration is very important, and a distinguishing feature of empirical bioethics, but we also need to think carefully about how we get there, and to date the methodological literature has paid relatively little attention to the significant amount of research that needs to be done in the lead up to integration.

## The framework

### Mapping, framing and shaping

The framework, which comprises three phases or stages, can be conveyed via a landscaping metaphor: mapping, framing, and shaping.

In the first, *mapping*, phase, the aim is to survey and get a sense of the general terrain. To flesh out the metaphor, we have a sense of where we are, and we have sense of what we want to do with the land (in the form of questions or issues we wish to explore) – but before we can landscape the terrain in front of us, we need to know what it is we have to work with. Are there hidden boulders? What kind of soil is there? Are there areas too dense and impenetrable to be worth attempting to shape? Are there natural lines that we can build into our design or do we have to excavate the lot and re-build from scratch? Essentially, before we start our project of landscaping the terrain, we need to examine what is out there and create a ‘map’ that will help us navigate and plan. In a research project, led by their research questions, the researcher(s) will seek to understand the “state of the art” and identify what is (not) known, specify gaps in the literature, identify further questions, and identify existing proposals for addressing such questions. This phase should enable the researcher(s) to work out what further work is now needed and (if needs be) hone their research questions and intended approach accordingly. As might be anticipated, this phase might involve some empirical inquiry, but it is likely to be literature-focused, analysing previous scholarship, opinions and data, which are drawn from the range of sources and disciplines that best suits the project in question.^1^

In the second, *framing*, phase, the aim is to explore specific areas of the mapped terrain that have been identified as either being in need of deeper exploration or unmappable from our current vantage point.^2^ In our metaphor, this is akin to commissioning specialist surveys to tell us, for example, what kind of bedrock is present, how stable certain areas are, whether there are any endangered species that must be protected – all of which will affect what we may want, or are able, to do with the land. In a research context, this will often have a focus on developing an understanding of how key issues are experienced (or “framed”) by relevant stakeholders. We may seek to frame, inter alia*,* questions, problems, experiences or possible solutions, but the common thread is that the issues are framed by the lived experience of relevant stakeholders. To build the metaphor, we are looking in-depth from multiple different angles to identify hidden tracks, perils, dips, rock formations or ravines that were not visible to us during the mapping stage but will affect what we are able to do with the land. Some of these may prove to be avoidable, some may be removable, and some may be fixed features of the landscape that we have no option but to accept and design around. Here, more finely-grained perspectival information is gathered – essentially from experienced travellers who have already traversed the terrain – which (again) might shed light on what is (not) known, reveal further questions, and/or indicate possible ways forward. This phase is empirical in orientation and, depending on the study and its research questions, researchers might seek to gather and analyse data from a variety of stakeholders in order to better understand the area and the perceptions and judgments of its occupants.

In the first two phases, the focus is on building an in-depth and intimate understanding of the terrain, so that we are in a position to determine how we want to shape it and how it can be shaped. It is descriptive (insofar as we describe what is there) but also critically normative (insofar as we analyse the features we describe to determine how strong, flexible and/or navigable they are).

In the third, *shaping*, phase, the aim is to seek to (re) shape the terrain, informed by the findings and analyses generated while mapping and framing. Equipped with – and attentive to – these different findings and the analysis thereof, the focus here is on formulating recommendations for ways forward. In our metaphor, this is the development of a landscaping design that will re-shape the terrain into its desired form. Armed with an intimate understanding and knowledge of the terrain, the designer can build a vision for what s/he wants, and explain why certain features have to be in certain places – sometimes for aesthetic reasons, sometimes for pragmatic reasons, and oftentimes aimed at an artful blending of the two. Sometimes, to reach the vision, a great deal of effort will be put into removing or overcoming an obstacle, but sometimes it may be more desirable or necessary to work around it or amend the vision to accommodate it. In research, this phase equates to the drawing of normative conclusions and, for the research to qualify as *empirical bioethics*, the researcher(s) will draw on an explicit empirical bioethics methodology, which will enable them to combine the different elements and explain how and why they accommodate the varying demands presented by theory and their empirical data. Here an additional metaphor might help: the methodology in question, whatever it happens to be, will provide a *bridge* between the more abstract, literature-led elements of the project (from the mapping phase), the empirically-led elements of the project (from the framing phase), and the normative, recommendations-focused elements of the project (in the shaping phase) [5]. This methodology must provide unifying structure, which shows how the different elements combine – much like the way a good landscaper will provide structure and themes to blend the different elements of the terrain together so that it is easily traversed.

### Illustrations

Whilst this articulation of our framework originated in Bristol’s Centre for Ethics in Medicine, we should note that it is not necessarily deployed in every project undertaken in that Centre. We also suspect that others may be doing something similar, albeit not articulating their work in quite this way. We therefore believe that the framework might well have wide relevance and utility because, even if it is recognised and already intuitively practiced, our framework provides a way of articulating the research phases in a clear and recognisable form. However, and certainly in the absence of empirical inquiry, we would not suggest that the framework can account for every form of empirical bioethics research, wherever it is undertaken.

Irrespective of the scope of its application, we nevertheless consider this framework to be useful because it is accommodating and pluralistic and, whichever precise approach is taken or whichever empirical bioethics methodology is applied (we will later refer to this as a necessary ‘bridging methodology’), it clearly indicates what we consider to be three key phases of empirical bioethics research.^3^ These positive attributes can be illustrated with reference to a selection of empirical bioethics projects that have been, or are being, undertaken by colleagues in the Bristol Centre, in which we have been involved as researchers, collaborators or supervisors. We note that our intention is not to critically examine the methodological choices made in these projects, but rather to illustrate how they fit with the “Mapping-Framing-Shaping” phases, despite their often very different methodological orientations.

Swift’s PhD project, which was completed in 2011, provides our first illustration [12–14]. Swift sought to explore the ethical dimensions of using “sham surgery” (or “placebo surgery”) control groups in the context of neurological research for Parkinson’s Disease. The three phases of her project align perfectly with the Bristol framework. First, she undertook a systematic literature review, exploring the ethics of sham surgery. Secondly, she undertook semi-structured interviews, using vignettes, with people with Parkinson’s Disease and their close family members. Thirdly, she combined the data and analysis from the first two phases to issue recommendations. To *bridge* these elements, Swift adopted Frith’s “symbiotic empirical ethics”, [15] which involved: setting out the circumstances (via the literature review); specifying theories and principles (via the literature, in particular Foster’s account of research ethics) [16]; using ethical theory as an analytic tool (i.e. using Foster’s framework to analyse the empirical data); theory building (using the empirical data to revise the relevant theory); and making normative judgments (bringing together the theory and the data).

Secondly, Birchley’s PhD project, which concluded in 2015, investigated the ethical aspects of “best interests” decision-making in the paediatric intensive care setting [17, 18]. Like Swift, Birchley aimed at establishing coherence between the theoretical and empirical aspects of his study. On Birchley’s account, his project involved the following (iterative) stages: undertaking a literature review to identify established theoretical accounts; gathering qualitative data from those with experience; developing his own considered moral judgments through critical review of the findings from the first two stages; seeking coherence between the preceding elements, using additional theory where necessary; transparently documenting all the stages; and repeating stages as necessary and airing the findings and analysis with relevant groups ([17], pp. 115ff). Like Swift’s work, this approach fits the Bristol framework, despite there being significant differences in the detail of the approach. For example, Birchley approached the literature reviews differently (undertaking a critical interpretive review of pertinent literatures, especially in ethics and law), interviewed healthcare professionals as well as patients and those close to them, and his *bridging methodology* was based on “reflective equilibrium” [19].

Thirdly, Morley’s project, which concluded in 2018, explored the concept of “moral distress” in nursing and aimed to provide a definition of moral distress in order to help clarify how it can be responded to. As in the previous projects, Morley aimed to achieve an integrated analysis, collecting empirical data about nurses’ experience of moral distress to inform a normative definition. The research broadly adopted feminist empirical bioethics as a *bridging methodology*, using “reflexive balancing” to integrate the conceptual and empirical elements and reach normative conclusions [6]. This began with a systematic literature review and narrative synthesis [20], which led to the development of a working definition of moral distress, but also identified a series of questions. These questions were then explored in a feminist interpretive phenomenological empirical investigation to capture the moral distress experience of UK nurses working in critical care. Finally, the working definition was refined in light of the empirical data and used to theorise a model of moral distress, which was then systematically challenged and revised through reflexive balancing [6], to develop an account of moral distress that was coherent and defensible. Although Morley’s methods, and theoretical orientation, differed from both Swift and Birchley, the phases of research similarly fit into the mapping (literature review), framing (empirical study) and shaping (theorising and developing a model) framework.

The framework does not only lend itself to PhD projects, in which a sole researcher is primarily responsible for the different elements of the study. As a final example, in autumn 2018, a team of researchers from the University of Bristol began work on a Wellcome Trust-funded collaborative project, “Balancing Best Interests in Healthcare, Ethics and Law (BABEL)” [10]. This five-year project is led by colleagues from the Bristol Centre for Ethics in Medicine and from Bristol’s Centre for Health, Law, and Society. The project comprises four work streams, each exploring different dimensions of “best interests” decision-making, involving a wide range of patients and professionals. As the project comprises ethical and legal elements, the researchers look not only to empirical bioethics research methodologies, but also to socio-legal studies, and they also seek to explore questions of methodology in a dedicated work stream. However, the project as a whole explicitly adopts the mapping, framing and shaping framework: literature review methods include “systematic reviews of reasons”, [11] empirical data are collected via various methods, and the overarching methodology, like Morley’s project, is based on Ives’ “reflexive balancing” [6]. This initially involves identifying the moral problem, and looking to theory, experience, or a mixture thereof. Thereafter, there is disciplinary-naïve inquiry into the problem i.e. exploring the literature and data to understand the problem and “find some basic value propositions, which can act as quasi-foundational boundary principles” ([6], p. 311). The final stage involves seeking overall coherence by systematically challenging the boundary principles and explaining why particular principles are accepted or rejected.

There are numerous other examples we could cite to further illustrate the flexibility of this framework. Amongst the other (previous and current) projects in our Centre to note are those which adopt the “critical applied ethics” [21] methodology, those which involve in-depth analysis of legal and disciplinary decisions in the framing phase, and those which anticipate including a consensus building exercise in the final shaping phase [22]. As such, the framework can help to delineate the different phases of empirical bioethics research, but doing so does not impose constraints on the researchers in terms of methodological orientation or the more specific methods adopted. As we have seen from these Bristol examples, literature reviews can take different forms, the empirical phase can involve various methods, and diverse “bridging” methodologies can be deployed (for example, “reflective equilibrium” or “symbiotic empirical ethics”).

### Limitations?

The framework appears to be usefully pluralistic and accommodating, but it might be objected that its breadth is a potential source of weakness and, conversely, that it is not actually as accommodating as it appears. We anticipate three associated questions, although we think each can be answered.

First, is this framework too accommodating i.e. too broadly applicable? If so, then what, if anything, does this framework add? Viewed superficially, the framework is potentially banal, as the three key phases appear to correspond with the approaches taken in a multitude of studies in a multitude of disciplines and fields. Many empirical studies in the social and health sciences will begin with a literature review, proceed to empirical data collection and analysis, and then conclude with the provision of recommendations.

One response to this first set of questions is that this similarity is a source of strength, as it should help to demystify – and potentially enhance the credibility of – empirical bioethics research for researchers operating in other disciplines and fields. As such, empirical bioethics research involves phases that will be familiar to those working elsewhere. But beyond this, secondly, empirical bioethics research remains a discrete endeavour. The key point of differentiation, which unites the entire endeavour of empirical bioethics research and is most apparent in the framework’s third phase, is how such research generates its recommendations. In short, empirical bioethics research involves distinctive methodologies that seek to bridge the abstract and the empirical to propose normative recommendations.

There are a multitude of such bridging methodologies [7], which provide some account of how the empirical and the normative can be integrated to generate solutions to ethical problems. Examples include the aforementioned symbiotic bioethics [15], critical applied ethics [21], and reflexive balancing [6], as well as particular varieties of reflective equilibrium [23] and alternative approaches like hermeneutic ethics [24]. These methodologies might be somewhat mystifying to outsiders (and even to some insiders), but they are what makes empirical bioethics research a distinct endeavour. The role of this bridging methodology is to show how the various elements of the research, undertaken across the mapping and framing phases, can be brought together and used (sometimes, but not always, alongside ethical theory), to develop normative claims in the shaping phase. As such, ‘Mapping-Framing-Shaping’ could describe many kinds of research endeavour. But when it is combined with a bridging methodology, it becomes empirical bioethics.^4^

Studies which do not attend (sufficiently) to this bridging element might resemble our account of empirical bioethics research in their phases, but they will likely best be considered – and judged as – something other than empirical bioethics research. Zoe Fritz’s – impressive – work on DNACPR (do not attempt cardiopulmonary resuscitation) decisions provides a useful illustration.

Fritz’s collaborative project, which formed the basis of her PhD [25], certainly appeared to proceed through – and, indeed, beyond – the three phases of the Bristol framework. First, she undertook literature reviews and ethical analysis of DNACPR decisions [26]. Secondly, she conducted a range of empirical work and related analysis, including a questionnaire study [27], observational study [28], combined observational and interview study [29], and an audit of practices [30]. Thirdly, she issued recommendations, which took the form of the UFTO (Universal Form of Treatment Options) [31]. These three phases echo the Bristol framework. Beyond these, Fritz also trialled and evaluated the UFTO [32], before playing a role in the development of a further proposal, ReSPECT (Recommended Summary Plan for Emergency Care and Treatment) [33], which is being further trialled and evaluated in different regions in the UK.

Fritz’s mixed-methods project broadly corresponds with the Bristol framework, although it is an open question whether it is strictly a project in empirical bioethics research as we earlier defined this. The missing ingredient appears to be an explicit account of the bridging methodology, which ties the different elements together and marks out a project as ‘empirical bioethics’. This might, of course, be attributable (at least in part) to journal conventions, since the articles were published separately. However, the omission also has a temporal explanation: Fritz’s work began in 2008, since which time there has been a significant expansion in the published accounts of empirical bioethics methodologies. Fritz has confirmed that there was no overarching, bridging methodology from the outset [34], although, as it proceeded, she drew on Dunn et al’s methodological proposals [35]. The lack of an explicit bridging methodology is not a criticism of Fritz’s work, which is carefully plotted and conducted, in line with the expectations associated with the different methods with (and thus fields in) which Fritz is working. Rather, this is simply an illustration of the point that the Bristol framework is not so broad that it encompasses virtually any project that combines literature-led and empirical limbs and seeks to issue recommendations: only those studies with the relevant, explicitly articulated, empirical bioethics bridging methodology will come within its remit.

The second and third questions take the opposite view, that the framework is too narrow. Secondly, then, is this framework limited to studies which involve qualitative, as opposed to quantitative, empirical research? Certainly, the Bristol Centre’s experience has been dominated by qualitative methods (albeit a variety of these). Our sense is that we are not alone in primarily tethering empirical bioethics research to qualitative inquiries: many of the different methodologies that are available seem also to focus on these types of study.

We are, nevertheless, clear that the Bristol framework accommodates quantitative work. Of course, the challenge – for the field as a whole – lies in devising appropriate bridging methodologies for combining normative reflections with quantitative data. Insights should be available from the literature on, for example, consensus-building models [36], although we suspect that such methodologies will require particular reflection on such contested issues as the role and weight of experts’ contributions [37]. We will leave these matters for elsewhere, save for emphasising that – provided that appropriate methodologies can be devised – it seems plausible that the direction of such research would still follow the three phases depicted in our framework.

Thirdly, and related to these reflections, is the framework limited to coherence-based, as opposed to consensus-based, approaches to empirical bioethics research? The examples we gave earlier all utilised some sort of coherence-based methodology. However, the framework and its three phases certainly do not exclude consensus-led approaches, but it might require some adaption depending on the bridging methodology employed. For example, a project might begin with literature reviews, which inform empirical data collection and analysis, and could then conclude with a consensus exercise, such as a DELPHI, whose key questions are informed by the previous phases [37]. Alternatively, a project may begin with a literature review and then may run the second and third phases in parallel – which may be so directed by various “dialogical” methodologies [7]. As previously noted, provided there is a relevant empirical bioethics methodology which links the three phases, such work can qualify as empirical bioethics research within the framework we outline.

## Conclusions

To briefly conclude, in this paper we have outlined our framework for empirical bioethics research, which we have articulated via a landscaping metaphor and illustrated with examples from our Centre’s research. The three phases – ‘Mapping-Framing-Shaping’ – can be applied, we argue, to empirical bioethics research generally without prescribing any specific methods or bridging methodology. It therefore might be useful to researchers who are in the process of planning projects, who need to find a way of describing the overarching shape of a project or programme of research in a way that will be familiar and acceptable to a range of disciplines, and yet retain the much needed flexibility to use whichever methods and empirical bioethics methodology is best suited to their research question(s).

In short, we offer this framework not to prescribe a singular way of carrying out empirical bioethics research, but as an example of an approach that we have found to work, and which may resonate usefully with others working in the field.

## Data Availability

Not applicable.

## Abbreviations

DNACPR: Do not attempt cardiopulmonary resuscitation
ReSPECT: Recommended Summary Plan for Emergency Care and Treatment
UFTO: Universal Form of Treatment Options

## Footnotes

1 This paper is concerned with empirical bioethics research specifically, but it would be a valid question to ask whether this kind of ‘mapping’ exercise is useful – or even essential – for any and all ethics research. Common sense would suggest that it is good – and essential – academic practice to have a good sense of what the existing literature says about a topic before writing about it, but there is still an important question to ask about whether this kind of exercise should – or should always be – systematic in nature. Sofear and Strech [11], for example, have argued in favour of a more systematic approach to literature reviews in ethics.

2 It has been helpfully pointed out by a reviewer of this paper that the term ‘framing’ has multiple connotations, and that we need to clarify precisely how we are using this term, especially for non-native English speakers. ‘Framing’, here, describes looking at a phenomenon from a particular vantage point, or through a particular lens, in order to see/understand it in a particular way. If we were to ‘frame’ a picture, for example, we place a physical barrier at the edges of the image to clearly demarcate the boundaries of where we should be looking and focus attention on the image. Similarly, in ‘framing’ an ethical issue we are – metaphorically – looking at the issue from a particular perspective and putting a ‘frame’ around it to focus our attention on that specific viewpoint and help us to see it more clearly.

3 It currently leaves aside what happens thereafter e.g. in generating impact and evaluating any proposals if adopted.

4 We are explicitly not, in this paper, evaluating different kinds of empirical bioethics ‘bridging’ methodologies.
